# SOFA is superior to MOD score for the determination of non-neurologic organ dysfunction in patients with severe traumatic brain injury: a cohort study

**DOI:** 10.1186/cc5007

**Published:** 2006-08-01

**Authors:** David Zygun, Luc Berthiaume, Kevin Laupland, John Kortbeek, Christopher Doig

**Affiliations:** 1 Department of Critical Care Medicine, University of Calgary, Calgary, Alberta, Canada; 2 Department of Clinical Neuroscience, University of Calgary, Calgary, Alberta, Canada; 3 Department of Medicine, University of Calgary, Calgary, Alberta, Canada; 4 Department of Community Health Sciences, University of Calgary, Calgary, Alberta, Canada; 5 Department of Surgery, University of Calgary, Calgary, Alberta, Canada

## Abstract

**Introduction:**

The objective of the present study was to compare the discriminative ability of the Sequential Organ Failure Assessment (SOFA) and Multiple Organ Dysfunction (MOD) scoring systems with respect to hospital mortality and unfavorable neurologic outcome in patients with severe traumatic brain injury admitted to the intensive care unit.

**Method:**

We performed a prospective cohort study at Foothills Medical Centre, the sole adult tertiary care trauma center servicing southern Alberta (population about 1.3 million). All patients aged 16 years or older with severe traumatic brain injury and intensive care unit length of stay greater than 48 hours between 1 May 2000 and 31 April 2003 were included. Non-neurologic organ dysfunction was measured using the SOFA and MODS scoring systems. Determination of organ dysfunction for each non-neurologic organ system was compared between the two systems by calculating the proportion of patients with SOFA and MOD component score defined organ failure. Consistent with previous literature, organ system failure was defined as a component score of three or greater.

**Results:**

The odds of death and unfavorable neurologic outcome in patients with SOFA defined cardiovascular failure were 14.7 times (95% confidence interval [CI] 5.9–36.3) and 7.6 times (95% CI 3.5–16.3) that of those without cardiovascular failure, respectively. The development of SOFA-defined cardiovascular failure was a reasonable discriminator of hospital mortality and unfavorable neurologic outcome (area under the receiver operating characteristic [ROC] curve 0.75 and 0.73, respectively). The odds of death and unfavorable neurologic outcome in patients with MOD-defined cardiovascular failure were 2.6 times (95% CI 1.24–5.26) and 4.1 times (95% CI 1.3–12.4) that of those without cardiovascular failure, respectively. The development of MOD-defined cardiovascular failure was a poor discriminator of hospital mortality and unfavorable neurologic outcome (area under the ROC curve 0.57 and 0.59, respectively). Neither SOFA-defined nor MOD-defined respiratory failure was significantly associated with hospital mortality.

**Conclusion:**

In patients with brain injury, the SOFA scoring system has superior discriminative ability and stronger association with outcome compared with the MOD scoring system with respect to hospital mortality and unfavorable neurologic outcome.

## Introduction

Multiple organ dysfunction syndrome is a major cause of death in multisystem intensive care unit (ICU) patients. Similar to all critically ill ICU patients, patients with life-threatening neurologic injury are at risk for development of multiple organ dysfunction syndrome. However, non-neurologic organ dysfunction has been described in patients with neurologic injury in the absence of the usual etiologic associations, namely infection or systemic traumatic injury. Therefore, severe neurologic injury represents an additional risk factor for the development of the multiple organ dysfunction syndrome. Importantly, the development of non-neurologic organ dysfunction, independent of the severity of neurologic injury, was recently associated with unfavorable outcome in patients with subarachnoid hemorrhage [1] and severe traumatic brain injury (TBI) [2].

Although several multiple organ dysfunction scoring systems [3] have been described, the Sequential Organ Failure Assessment (SOFA; Table 1) score [4] and the Multiple Organ Dysfunction (MOD; Table 2) score [5] are most commonly applied. However, until recently neither score was validated in neurologic critical illness in a population-based study [2]. Furthermore, the performance of these scores may be affected by the therapy used to support the cerebral circulation. The MOD cardiovascular component score is calculated based on the pressure-adjusted heart rate (PAR). Theoretically, because it is therapy independent, PAR is advantageous in this population in which cerebral perfusion pressure management is the standard of care. The SOFA cardiovascular component is calculated based on mean arterial pressure and inotrope requirement. Despite a recent study in general systems ICU patients suggesting a stronger relationship of the SOFA cardiovascular component with mortality compared with the MOD cardiovascular component score [6], the therapy dependence of this SOFA component may not allow it to discriminate between cerebrovascular support and cardiovascular failure in patients with severe neurologic injury.

The objective of the present study was to describe and compare the non-neurologic SOFA and MOD component scores' association with and ability to discriminate outcome in a cohort of patients with severe TBI.

## Materials and methods

The present study was a cohort study comprising data merged from two prospectively collected databases. Patients with severe TBI were identified from the Trauma Services database maintained by the Division of Trauma, Department of Surgery at Foothills Medical Centre (FMC), Calgary, Alberta, Canada. The Department of Critical Care Medicine TRACER database prospectively records organ dysfunction (SOFA and MOD) scores on all patients admitted to the ICU for each day of their ICU stay and mortality status. Ethical review and approval was attained from the regional ethics review board.

In the Calgary Health Region, adult trauma services are regionalized to the FMC, which is the sole adult tertiary care trauma center servicing southern Alberta, Canada (population about 1.3 million). All adult patients (≥16 years of age) with severe TBI admitted to the ICU of FMC during the period from 1 May 2000 to 30 April 2003 with an ICU length of stay (LOS) greater than 48 hours were included. Severe TBI was defined as a TBI resulting in at least one of the following: an initial resuscitated (systolic blood pressure >90 mm Hg and arterial oxygen saturation >90%) Glasgow Coma Score (GCS) of 8 or less at first contact with medical services; a post-resuscitation GCS at presentation to the trauma centre of 8 or less in the absence of sedation; need for intracranial pressure monitoring; or the presence of a clinical herniation syndrome as verified by the attending physician.

Management of patients was protocolized with a cerebral perfusion pressure goal of 70 mmHg and intracranial pressure goal of <20 mmHg. Initial optimization of cerebral hemodynamics was accomplished with sedation (propofol and morphine), normocapnia, normothermia, normoglycemia, and euvolemia. Briefly, elevations in intracranial pressure were managed sequentially with paralysis, mannitol, mild hypothermia, and mild hyperventilation (arterial carbon dioxide tension 30–34 mmHg) under jugular saturation monitoring guidance. Barbiturate therapy or decompressive craniectomy was considered for refractory intracranial pressure.

As described previously [7], the SOFA and MOD scores were collected daily based on the recommendations in the original publications [8,9]. An electronic patient information system (Quantitative Sentinel [QS]; GE-Marquette Medical Systems Inc. Milwaukee, WI. USA) interfaced to all bedside devices recorded physiologic data, and these data were validated (accepted by the system) by nursing or respiratory therapy staff on an at least hourly basis by examining the degree to which they were representative and sensible. An HL-7 interface with the regional laboratory information system (Cerner PathNet Classic version 306 [Kansas City, MO, USA]) was utilized to collect all laboratory data.

Two programs were developed in Visual Basic (Microsoft VBL; Microsoft Corporation, Seattle, WA, USA) to examine all physiologic and laboratory values in each 24 hour period, measured daily from 00:00 hours to 23:59 hours. For the SOFA score, one Visual Basic program determined the most abnormal value for each parameter. The program then calculated the appropriate SOFA value (range 0–4), which was then exported to a local longitudinal ICU database known as TRACER (Microsoft Access; Microsoft Corporation). Missing values were replaced between a preceding and subsequent value with the lower of the two scores. In the absence of a preceding or subsequent value, the score was calculated at zero. In the second Visual Basic program, the least abnormal value at 07:00 ± two hours was used to calculate the appropriate MOD score. The calculation of each component system value and the total values for both SOFA and MOD scores were manually checked by one of the investigators (CD) for their accuracy by comparing them with the laboratory or physiologic data recorded in the QS system over a one month period (683 patient-days) before the start of the study; no errors were found in the calculation of either score. Patient demographics, injury details, Injury Severity Score, Abbreviated Injury Scale, and post-resuscitation GCS were included in the Trauma Services database. ICU and hospital LOS were included in the TRACER database. Glasgow Outcome Scores (GOS) were determined at hospital discharge.

### Data analysis

Descriptive statistics and box plots were used to analyze each variable separately. Analyses of continuous, normally distributed variables within and between groups were undertaken using the appropriate Student's *t *test. Non-normally distributed continuous variables were analyzed using the Mann-Whitney *U *test. Categorical variables were analyzed using Fisher's exact test. *P *< 0.05 was considered statistically significant. All statistical tests were two sided.

Determination of organ dysfunction for each non-neurologic organ system was compared between the two systems by calculating. Consistent with previous literature, organ system failure was defined as a component score of three or greater. The proportion of patients who did not survive to hospital discharge was calculated for each level of dysfunction within each component score and the results for SOFA and MOD scores were compared. Organ systems with discrepant results were further analyzed by calculating the odds ratio (OR) for hospital mortality of SOFA-defined or MOD-defined organ failure. Ability to discriminate hospital mortality was judged by calculating the area under the receiver operating characteristic (ROC) curve. GOS was dichotomized into favorable outcome (GOS 4, 5) and unfavorable outcome (GOS 1, 2, 3), and a similar analysis was repeated.

## Results

### Patient characteristics

A total of 209 patients were identified as having sustained a severe TBI and required at least 48 hours of ICU care during the study period. The characteristics of these patients are detailed in Table 3.

The percentages of patients with SOFA and MOD component score defined organ failure are presented in Table 4. For four of the five non-neurologic organ systems, SOFA component scores identified organ failure in a higher proportion of patients.

The relationship of hospital mortality and component SOFA and MOD scores are presented in Table 5. Mortality increased with increasing SOFA cardiovascular component score. However, there was no significant difference in mortality between MOD cardiovascular component scores greater than zero. The distribution of patients differed dramatically between the SOFA and MOD cardiovascular component scores. The majority of patients (105) were identified by SOFA cardiovascular component score as having the most severe degree of cardiovascular dysfunction, whereas the MOD cardiovascular component score determined almost half of the patients (100) as having normal cardiovascular function. Patients who developed SOFA-defined cardiovascular failure were at significantly greater risk for death than those patients who did not (OR 14.7, 95% confidence interval [CI] 5.9–36.3; *P *< 0.001). The development of SOFA defined cardiovascular failure was a reasonable discriminator of hospital mortality (area under the ROC curve 0.75). Those patients who developed MOD-defined cardiovascular failure had a slightly increased risk for hospital mortality (OR 2.6, 95% CI 1.24–5.26; *P *= 0.01). The development of MOD-defined cardiovascular failure was a poor discriminator of hospital mortality (area under the ROC curve 0.57). When examining vasopressor use and its comparison to MOD-defined cardiovascular failure (vasopressor independent variable), there were 655 patient-days on which vasopressors were used. Of these days, 611 (93%) were not classified as cardiovascular failure by MOD score. However, for those patients requiring vasopressors, MOD-defined cardiovascular failure was not associated with hospital mortality (*P *= 0.42). This suggests MOD defined cardiovascular failure is a poor discriminator of outcome rather than SOFA overcalling cardiovascular failure due to vasopressor use for cerebrovascular support.

**Figure 1:**
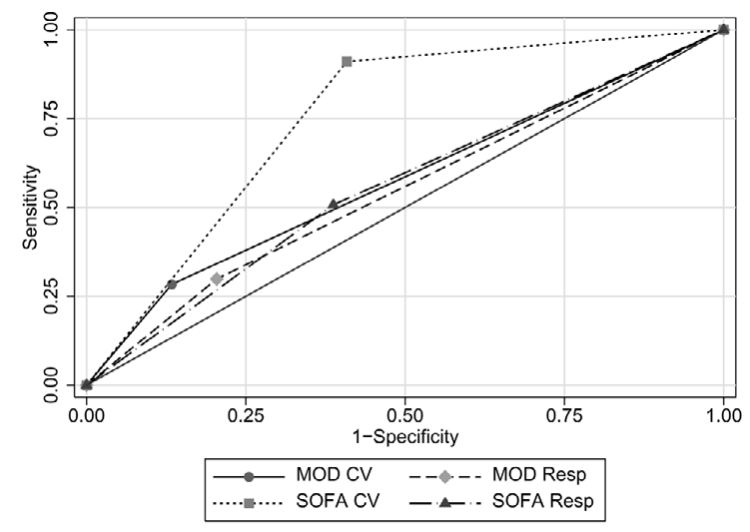
Area under the ROC curve for hospital mortality by SOFA and MOD score organ system failure. CV, cardiovascular; MOD Multiple Organ Dysfunction; ROC, receiver operating characteristic; SOFA, Sequential Organ Failure Assessment.

In general, an increasing SOFA respiratory component score was associated with increasing mortality. This was not the case for the MOD respiratory component score. In fact, the highest MOD respiratory component score was associated with the lowest mortality. Respiratory organ failure defined by either score was not significantly associated with increased risk for death before hospital discharge. A graphical representation of the area under the ROC curve results is presented in Figure 1 for the each score's cardiovascular and respiratory components. For the renal, coagulation, and hepatic component scores, there was little difference between the SOFA and MOD scoring systems.

**Figure 2:**
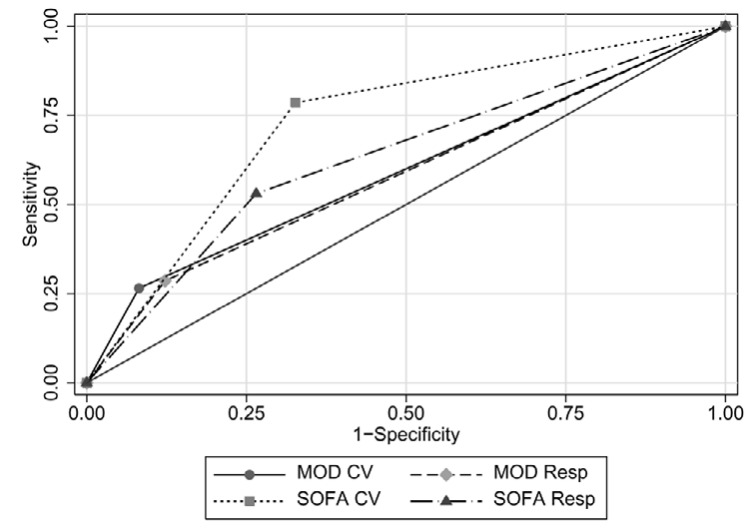
Area under the ROC curve for unfavorable urologic outcome by SOFA and MOD score organ system failure. MOD Multiple Organ Dysfunction; ROC, receiver operating characteristic; SOFA, Sequential Organ Failure Assessment.

The relationships between dichotomized neurologic outcome and component SOFA and MOD scores are presented in Table 6. Similar to the data regarding hospital mortality, the distribution of patients and proportion of patients with unfavorable neurologic outcome differed between SOFA and MOD cardiovascular component cardiovascular scores. Developing cardiovascular failure as defined by SOFA was associated with a greater risk for unfavorable neurological outcome (OR 7.6, 95% CI 3.5–16.3; *P *< 0.001) than developing MOD-defined cardiovascular failure (OR 4.1, 95% CI 1.3–12.4; *P *= 0.006). SOFA-defined cardiovascular failure was a better discriminator of dichotomized neurologic outcome than MOD-defined cardiovascular failure (area under the ROC curve 0.73 versus 0.59). Graphical representation of the area under the ROC curve results is presented in Figure 2 for the each score's cardiovascular and respiratory components. For the renal, coagulation, and hepatic component scores, there was little difference between the SOFA and MOD scoring systems.

Patients were further categorized as having SOFA-defined and MOD-defined cardiovascular failure, SOFA-defined but not MOD-defined cardiovascular failure, MOD-defined but not SOFA-defined cardiovascular failure, and patients without SOFA-defined or MOD-defined cardiovascular failure. This categorization was tabulated in association with hospital mortality, which was the most robust end-point of the study. A similar process was repeated for the respiratory component scores. The results for cardiovascular failure are presented in Table 7. Patients with SOFA-defined and MOD-defined cardiovascular failure suffered the greatest hospital mortality, but this was not significantly different from those patients with SOFA-defined but not MOD-defined cardiovascular failure. This suggests little additive contribution of MOD-defined cardiovascular failure if patients have SOFA-defined cardiovascular failure. Furthermore, all five patients with MOD-defined but not SOFA-defined cardiovascular failure survived. This mortality was not significantly different from that in those patients without cardiovascular failure. Age and post-resuscitation GCS was not significantly different among the four categories.

The results for respiratory failure are also presented in Table 7. MOD-defined respiratory failure did not occur in the absence of SOFA-defined respiratory failure. Patients with SOFA and MOD-defined respiratory failure suffered the greatest hospital mortality but this was not significantly different from that in those patients with SOFA-defined but not MOD-defined respiratory failure. This again suggests little additive contribution of MOD-defined organ failure if patients have SOFA-defined failure. Age and post-resuscitation GCS were not significantly different among the four categories.

## Discussion

Brain injury is a pro-inflammatory state that may be an important mechanism of organ dysfunction and ultimately multiple organ dysfunction syndrome [10-14]. Non-neurologic organ dysfunction is common in patients with traumatic and nontraumatic neurologic injury [1,2]. Organ failure is independently associated with mortality and poor neurologic outcome in this subset of patients [1,2]. Therefore, it is of paramount importance to have a valid and reliable organ dysfunction classification system for both clinical and research purposes. The SOFA and MOD scores have been shown to discriminate outcome in multisystem ICU patients [4,5].

In this cohort of patients, the proportion of patients with renal, hepatic, and hematologic failure was small. However, the proportions of patients with SOFA-defined cardiovascular and respiratory failure were 56% and 43%, respectively. MOD score defined cardiovascular and respiratory failure occurred in 18% and 23% of patients, respectively. This discrepancy may be explained by an underestimation of organ failure by the MOD score when these proportions are compared with the incidence of cardiovascular and respiratory failure stated in the literature [15,16]. Given that there were few patients with renal, hepatic, and hematologic failure, the ability to discriminate outcome in this cohort of neurocritical care patients will be a function of the cardiovascular and respiratory component scores of the MOD and SOFA scoring systems.

These data suggest that SOFA-defined cardiovascular failure has superior discriminative ability with respect to hospital mortality. For those with cardiovascular failure, the unadjusted odds of death in hospital were 14.7 times that of patients with normal cardiovascular function. The corresponding area under the ROC curve was 0.76, as compared with 0.57 for those with MODS-defined cardiovascular failure. In a prospective multicenter study, Moreno and colleagues [17] evaluated the ability of the maximum SOFA score to discriminate ICU outcome. The authors also evaluated the discriminative ability of each individual component score. The study population consisted of 1,449 patients admitted to a multisystem ICU with ICU LOS greater than 48 hours. In a multivariable logistic regression model, the cardiovascular component was associated with the highest contribution to outcome (OR 1.68). Peres Bota and colleagues [6] reported similar findings in their assessment of the ability of the maximum SOFA and MOD scores to discriminate outcome in a mixed medical-surgical ICU. The area under the ROC curve was 0.821 for the cardiovascular component of the SOFA scoring system, as compared with 0.750 for the same component of the MOD scoring system. The difference was even more pronounced when patients with shock were considered. The areas under the ROC curves were 0.806 and 0.640, respectively, with the SOFA cardiovascular component having superior discriminative ability.

The difference between the two systems is that in the MOD system, PAR is used to calculate the cardiovascular component whereas in the SOFA system the calculation stems from the mean arterial pressure as well as doses of vasoactive and inotropic agents. Marshall and colleagues [5] selected PAR because this variable was treatment independent. Although there is value in this feature, a significant caveat is that a situation may arise in which two patients have similar PAR scores but one of them may be on large doses of vasoactive medications whereas the other does not require blood pressure support. In the present study, the stronger association with mortality of SOFA-defined cardiovascular failure suggests this SOFA cardiovascular component score does not merely reflect therapeutic intervention in the form of blood pressure augmentation to maintain cerebral blood flow.

Surprisingly, neither SOFA-defined nor MOD-defined respiratory failure was significantly associated with increased hospital mortality. In a mixed medical-surgical ICU population, Moreno and colleagues [17] found that SOFA-defined respiratory dysfunction made an important relative contribution to ICU outcome (OR 1.176). Furthermore, Bratton and coworkers [18] performed a retrospective study of 1,030 patients registered in the Traumatic Coma Databank. Twenty per cent of patients in this group developed acute lung injury (ALI). Six months after injury, the acute GCS adjusted odds of poor outcome (death or vegetative survival) in those with ALI was 2.8 times (95% CI 1.9–5.6) that of patients without ALI. Holland and colleagues [15] investigated the effect of respiratory dysfunction on outcome in 137 patients with isolated head injury who were mechanically ventilated for at least 24 hours, and found 31% of patients met criteria for ALI. The patients who developed ALI had a significantly greater mortality than did those without ALI (38% versus 15%; *P *= 0.004).

A possible explanation for the lack of discriminative ability of the respiratory component scores in our study is that it was underpowered to detect such differences. Alternatively, in a large database ICU patients, Zimmerman and coworkers [19] found that a continuous physiologic measure is a more sensitive and accurate method for describing patients and estimating outcome than total scores or counting the number of organ system failures. Furthermore, a weakness of both scoring systems with respect to the respiratory component is they fail to account for treatment variables such as mean airway pressure and/or positive end-expiratory pressure.

As was the case with hospital mortality, the discriminative ability of the cardiovascular component of the SOFA scoring system was superior with respect to poor neurologic outcome. With cardiovascular failure as defined by the SOFA scoring system, the odds of unfavorable neurologic outcome were 7.6 times those in patients with preserved cardiovascular function. The corresponding area under the ROC curve was 0.73, as compared with 0.59 for those with MODS-defined cardiovascular failure. To our knowledge, this is the first comparison of both scoring systems attempting to discriminate unfavorable outcomes in patients with severe TBI.

Although organ dysfunction scores can provide potentially useful prognostic information because they have been validated against survival, these scores have not been developed for quantitative mortality prediction [20]. Organ dysfunction scores are more commonly used for descriptive purposes. In addition, these scores may be useful to adjust for baseline characteristics, to control for time-dependent changes, and to compare organ dysfunction between groups directly as a secondary outcome in trials. Measurement of organ dysfunction may increase our knowledge of mechanisms by which interventions exert their effect [20]. Organ dysfunction scores may be utilized clinically for the monitoring of therapeutic interventions. The need for this monitoring in neurotrauma was highlighted by Roberston and colleagues [21], who found a fivefold increase in the occurrence of adult respiratory distress syndrome in a group of patients with TBI managed with a cerebral blood flow targeted protocol.

There are limitations to this study that require discussion. Information regarding the acquisition of infection was not recorded in this group of patients. Pneumonia is a frequent complication of severe TBI and may be an independent predictor of mortality [22]. As such, it is difficult to exclude the possibility that the presence of pneumonia influenced our results. It is also noteworthy that there were data missing with respect to dichotomized GOS. It is plausible that these missing data favored one of the dichotomized neurologic outcomes. In addition, classification of neurologic outcome was determined by chart review and was not performed at a standardized time after injury but rather at hospital discharge. It is important to note that patients with severe TBI can improve over time after hospital discharge. It is possible that the differential timing of neurologic outcome might have had an impact on results for this end-point

## Conclusion

In patients with brain injury, the SOFA scoring system has superior discriminative ability and stronger association with outcome compared with the MOD scoring system with respect to hospital mortality and unfavorable neurologic outcome.

## Key messages

• Non-neurologic organ dysfunction is common in patients with traumatic neurologic injury and it is independently associated with mortality and poor neurologic outcome.

• It is therefore of paramount importance to have a valid and reliable organ dysfunction classification system for both clinical and research purposes.

• These data reveal that in patients with severe TBI, the SOFA scoring system has superior discriminative ability and stronger association with outcome than does the MOD scoring system with respect to hospital mortality and unfavorable neurologic outcome.

## Abbreviations

CI = confidence interval; FMC = Foothills Medical Centre; GCS = Glasgow Coma Scale; GOS = Glasgow Outcome Score; ICU = intensive care unit; MOD = Multiple Organ Dysfunction; OR = odds ratio; PAR = pressure-adjusted heart rate; ROC = receiver operating characteristic; SOFA = Sequential Organ Failure Assessment; TBI = traumatic brain injury.

## Competing interests

The authors declare that they have no competing interests.

## Authors' contributions

DZ, KL, JK, and CD designed the study. DZ and LB performed the analysis and wrote the manuscript. All authors edited and approved the final manuscript.

## Figures and Tables

**Table 1:** SOFA score

Organ system	Score				
	
	0	1	2	3	4
Respiratory: PaO_2_/FiO_2_	>400	≤400	≤300	≤200	≤100
Renal: creatinine (μmol/l)	≤110	110–170	171–299	300–440; urine output ≤500 ml/day	>440; urine output <200 ml/day
Hepatic: bilirubin (μmol/l)	≤20	20–32	33–101	102–204	>204
Cardiovascular: hypotension	No hypotension	MAP <70 mmHg	Dopamine ≤5^a^, dobutamine (any dose)	Dopamine >5^a ^or epinephrine ≤0.1^a ^or norepinephrine ≤0.1^a^	Dopamine >15^a ^or epinephrine >0.1^a ^or norepinephrine >0.1^a^
Hematologic: platelet count	>150	≤150	≤100	≤50	≤20
Neurologic: Glasgow Coma Scale score	15	13–14	10–12	6–9	<6

^a^Adrenergic agents administered for at least one hour (doses given are in μg/kg per minute). FiO_2_, fractional inspired oxygen; MAP, mean arterial pressure; PaO_2_, arterial oxygen tension; SOFA, Sequential Organ Failure Assessment.

**Table 2:** MOD score

Organ system	Score				
	
	0	1	2	3	4
Respiratory: PaO_2_/FiO_2_	>300	226–300	151–225	76–150	≤75
Renal: creatinine (μmol/l)	≤100	101–200	201–350	251–500	>500
Hepatic: bilirubin (μmol/l)	≤20	21–60	61–120	121–240	>240
Cardiovascular: PAR^a^	<10.0	10.1–15	15.1–20.0	20.1–30.0	>30.0
Hematologic: platelet count	>120	81–120	51–80	21–50	≤20
Neurologic: Glasgow Coma Scale score	15	13–14	10–12	7–9	≤6

^a^PAR is the product of the heart rate and the ratio of the right atrial pressure to the mean arterial pressure. FiO_2_, fractional inspired oxygen; MOD, Multiple Organ Dysfunction; PaO_2_, arterial oxygen tension; PAR, pressure-adjusted heart rate.

**Table 3:** Patient characteristics

Patient characteristic	Value
Number	209
Age (years; median [range])	36 (16–90)
Male (%)	78%
Injury Severity Score (mean ± SD)	32.6 ± 10.8
Mechanism of injury	
Motor vehicle collision	50%
Fall	33%
Assault	5%
Suicide	3%
Pedestrian versus motor vehicle	5%
Bicycle collision	1%
Snowboarding/skiing	1%
Unknown	1%
Post-resuscitation GCS score (median [interquartile range])	5 (3–7)
Admission APACHE II score (mean ± SD)	18.5 ± 6.4
Patients with following injuries on head CT (%)	
Subdural hematoma	54%
Extradural hematoma	16%
Subarachnoid hemorrhage	55%
Diffuse axonal injury	31%
Intraventricular hemorrhage	32%
Parenchymal hematoma	28%
Patients with maximum AIS ≥3 for following systems (%)	
Chest	87%
Abdomen/pelvic contents	39%
Pelvis/extremities	63%
ICU length of stay (median [IQR])	7 (3–13)
Hospital length of stay (median [IQR])	19 (6–50)
Hospital mortality (%)	32%

AIS, Abbreviated Injury Scale; APACHE, Acute Physiology nd Chronic Health Evaluation; CT, computed tomography; GCS, Glasgow Coma Scale; ICU, intensive care unit; IQR, interquartile range; SD, standard deviation.

**Table 4:** Percentage of patients with component score defined organ failure

Component score	SOFA	MOD
Cardiovascular	56%	18%
Respiratory	43%	23%
Coagulation	6%	4%
Renal	0.5%	0.5%
Hepatic	1%	0%

MOD, Multiple Organ Dysfunction; SOFA, Sequential Organ Failure Assessment.

**Table 5:** Relationship of survival status at hospital discharge and component SOFA and MOD scores

Maximum SOFA component score	Proportion of nonsurvivors	*n*	Maximum MOD component score	Proportion of nonsurvivors	*n*
CV component score			CV component score		
0	0	20	0	0.17	100
1	0.09	70	1	0.43	37
2		0	2	0.44	34
3	0.43	14	3	0.52	21
4	0.53	105	4	0.47	17
Respiratory component score			Respiratory component score		
0	0.17	6	0	0.31	39
1	0.21	24	1	0.32	57
2	0.30	90	2	0.27	64
3	0.28	57	3	0.44	41
4	0.56	32	4	0.25	8
Coagulation component score			Coagulation component score		
0	0.30	93	0	0.28	134
1	0.24	58	1	0.32	44
2	0.39	46	2	0.43	23
3	0.5	10	3	0.57	7
4	1.0	2	4	1.0	1
Renal component score			Renal component score		
0	0.30	195	0	0.31	193
1	0.5	10	1	0.38	13
2	0.67	3	2	0.5	2
3	1.0	1	3	1.0	3
4		0	4		0
Hepatic component score			Hepatic component score		
0	0.32	180	0	0.32	192
1	0.33	24	1	0.33	15
2	0.33	3	2	0.50	2
3	0.5	2	3		
4		0	4		

CV, cardiovascular; MOD, Multiple Organ Dysfunction; SOFA, sequential organ failure assessment.

**Table 6:** Association of dichotomized neurological outcome and component SOFA and MOD scores

Maximum SOFA component score	Proportion with unfavorable outcome	*n*	Maximum MOD component score	Proportion with unfavorable outcome	*n*
CV component score			CV component score		
0	0.27	11	0	0.49	65
1	0.42	43	1	0.70	27
2		0	2	0.84	25
3	0.70	10	3	0.93	15
4	0.84	83	4	0.80	15
Respiratory component score			Respiratory component score		
0	0.33	3	0	0.50	30
1	0.37	19	1	0.66	38
2	0.63	60	2	0.67	45
3	0.74	39	3	0.86	29
4	0.88	26	4	0.60	5
Coagulation component score			Coagulation component score		
0	0.62	68	0	0.62	94
1	0.51	39	1	0.66	32
2	0.91	32	2	0.93	15
3	0.83	6	3	0.80	5
4	1.0	2	4	1.0	1
Renal component score			Renal component score		
0	0.65	135	0	0.66	137
1	0.78	9	1	0.75	8
2	1.0	2	2	1.0	1
3	1.0	1	3	1.0	1
4		0	4		0
Hepatic component score			Hepatic component score		
0	0.67	128	0	0.66	136
1	0.63	16	1	0.70	10
2	0.50	2	2	1.0	1
3	1.0	1	3		0
4		0	4		0

CV, cardiovascular; MOD, Multiple Organ Dysfunction; SOFA, sequential organ failure assessment.

**Table 7:** Relationships between SOFA-defined and MOD-defined organ failure and mortality

Cardiovascular failure defined by	*n*	Hospital mortality	Respiratory failure defined by	*n*	Hospital mortality
SOFA and MOD	33	56%	SOFA and MOD	49	41%
SOFA not MOD	86	49%	SOFA not MOD	40	35%
MOD not SOFA	5	0%	MOD not SOFA	0	
Neither	85	7%	Neither	120	28%

MOD, Multiple Organ Dysfunction; SOFA, sequential organ failure assessment. CV, cardiovascular; MOD, Multiple Organ Dysfunction; SOFA, sequential organ failure assessment.
